# Cellular signaling modulated by miRNA-3652 in ovarian cancer: unveiling mechanistic pathways for future therapeutic strategies

**DOI:** 10.1186/s12964-023-01330-x

**Published:** 2023-10-16

**Authors:** Komal Imran, Muhammad Javed Iqbal, Rameesha Abid, Muhammad Mushtaq Ahmad, Daniela Calina, Javad Sharifi-Rad, William C. Cho

**Affiliations:** 1 Department of Biotechnology, Faculty of Sciences, University of Sialkot, Sialkot, Pakistan; 2 Department of Microbiology, Quaid-i-Azam University, Islamabad, Pakistan; 3 Department of Allied Health Sciences, International Institute of Science, Art and Technology, Gujranwala, Pakistan; 4 Department of Clinical Pharmacy, University of Medicine and Pharmacy of Craiova, 200349 Craiova, Romania; 5 Facultad de Medicina, Universidad del Azuay, Cuenca, Ecuador; 6 Department of Clinical Oncology, Queen Elizabeth Hospital, Kowloon, Hong Kong

**Keywords:** Ovarian cancer, miRNA-3652, KEGG, EMT, CDK

## Abstract

MicroRNAs (miRNAs) are small non-coding RNA molecules that play pivotal roles in regulating gene expression and have been implicated in the pathogenesis of numerous cancers. miRNA-3652, though relatively less explored, has recently emerged as a potential key player in ovarian cancer's molecular landscape. This review aims to delineate the functional significance and tumor progression role of miRNA-3652 in ovarian cancer, shedding light on its potential as both a diagnostic biomarker and therapeutic target. A comprehensive literature search was carried out using established databases, the focus was on articles that reported the role of miRNA-3652 in ovarian cancer, encompassing mechanistic insights, functional studies, and its association with clinical outcomes. This updated review highlighted that miRNA-3652 is intricately involved in ovarian cancer cell proliferation, migration, and invasion, its dysregulation was linked to altered expression of critical genes involved in tumor growth and metastasis; furthermore, miRNA-3652 expression levels were found to correlate with clinical stages, prognosis, and response to therapy in ovarian cancer patients. miRNA-3652 holds significant promise as a vital molecular player in ovarian cancer's pathophysiology. Its functional role and impact on tumor progression make it a potential candidate for diagnostic and therapeutic applications in ovarian cancer. Given the pivotal role of miRNA-3652 in ovarian cancer, future studies should emphasize in-depth mechanistic explorations, utilizing advanced genomic and proteomic tools. Collaboration between basic scientists and clinicians will be vital to translating these findings into innovative diagnostic and therapeutic strategies, ultimately benefiting ovarian cancer patients.

Video Abstract

**Supplementary Information:**

The online version contains supplementary material available at 10.1186/s12964-023-01330-x.

## Introduction

Ovarian Cancer (OC) constitutes one of the most lethal gynecological malignancies that threatens women’s health worldwide [72]. Ovarian malignant tumours have the highest mortality rate among all gynecological tumours in developed countries. According to estimates from the Centers for Disease Control and Prevention (CDC) and the American Cancer Society, ovarian cancer (OC) ranks as the second most common gynecological cancer in the United States. In 2021 alone, it is estimated that more than 21,410 women will be diagnosed with this disease [65]. Furthermore, the mortality rate is alarmingly high, exceeding 50%, with a high percent women expected to die from ovarian cancer in the same year [2, 11]. One significant challenge in managing ovarian cancer is the limited rate of early diagnosis. This is largely due to the absence of effective, sensitive diagnostic methods [13]. Additionally, the high mortality rate is exacerbated by the frequent occurrence of drug resistance and high rates of recurrence [11, 39]. Several studies have highlighted the prognostic importance of miRNA-3652 in ovarian cancer [20]; existing literature shows that high/low levels of miRNA-3652 are associated with poorer/better survival outcomes [62]. These findings suggest that miRNA-3652 could serve as a potential prognostic biomarker and highlight the urgent need for further studies aimed at understanding the mechanistic pathways modulated by this microRNA [20, 62]. A recent research led aimed to enhance the prediction of survival rates in ovarian cancer patients through the identification of specific miRNA markers [62]. Utilizing a unique algorithm known as OV-SURV, which combines support vector regression with a dual-objective genetic algorithm for feature extraction, the team scrutinized miRNA expression and survival statistics from 209 patients sourced from The Cancer Genome Atlas [62]. The algorithm exhibited strong predictive capabilities, reflected by a mean correlation coefficient of 0.77 and a mean absolute error rate of only 0.69 years during tenfold cross-validation. Key miRNAs like hsa-let-7f, hsa-miR-1237, and hsa-miR-98 were found to have a substantial impact on survival outcomes. Furthermore, pathway analysis pinpointed that specific groups of these miRNAs are predominantly involved in fatty acid production and breakdown processes [62]. The precise cause of OC is still uncertain, but specific risk and contributing factors, including increased age, ovulation, hormonal imbalance, cytokines, environmental factors, and genetic predisposition, have been identified. Aberrant transcriptional regulations are already known to have their role in the onset of different types of cancers, including OCs [53]. Despite advancements in generating cancer genome data, the biology and mechanisms of ovarian cancers (OCs) are still not fully understood. miRNAs, which are small non-coding RNAs of approximately 22 nucleotides in length, are involved in post-transcriptional regulation. These RNAs significantly regulate various cellular processes, such as cell growth, tissue differentiation, and apoptosis. The well-established role of several miRNAs in carcinogenesis is their ability to target specific mRNAs [25]. Alterations of miRNAs expression patterns are found to be associated with several diseases, including cancers. Different miRNAs are found to be involved in OC pathogenesis, such as miR-200, miR-506, miR-183, miR-20 and many more [29, 38, 78]. Additionally, alterations in the abundance of miRNA had been noted in patients with OC in many previous studies compared to healthy individuals [8, 26]. Novel targets in the research line are required as momentum for the diagnosis, treatment, and prognosis of OC. To this end, the expression analysis of miRNAs can emerge as a new hope regarding the management of OC. Therefore the correlation between aberrant expression of miRNAs and OC-promoting genes regulated pathways can provide new insights into the clinical manifestation, diagnosis and treatment of OC [49, 79]. Expression analysis has shown that the plasma and serum of ovarian cancer (OC) patients display downregulation of miRNA-3625 [28, 40]. This downregulation is associated with dysregulation of tumor-suppressive genes and overexpression of tumor oncogenes. Intriguingly, the OC patients that are resistant to front-line chemotherapeutic agents against OC [28] also show downregulation of mOC-resistant C-resistant cells exhibiting the epigenetic repairing of miRNA-3625 demonstrated to enhance the potency of therapeutic agents [14]. Overall, most of the reported studies on miRNAs were mainly focused on evaluating the abundance of miRNA along with speculations about their role in causing OC. No study, according to the best of our knowledge, has yet reported the concise mechanism and/or role of miRNA-3652 in causing OC. The study has addressed the issue since it offers thorough evaluations and potentially insightful information on the miRNA-3652 targeted genes and their function in controlling signalling pathways that influence malignant cell behaviour. For miRNA-3652 target sites’ recognition and verification in ovarian cancer, we have used a stereotypical and arduous approach. The study adopts a pathway-oriented method to pinpoint the relevant factors involved in cancer growth and describes the signalling cascades associated with ovarian cancer, thus paving the way for in-depth mechanistic studies and designing futuristic therapeutic strategies.

## Methodology

### miRNA selection

**Fig. 1:**
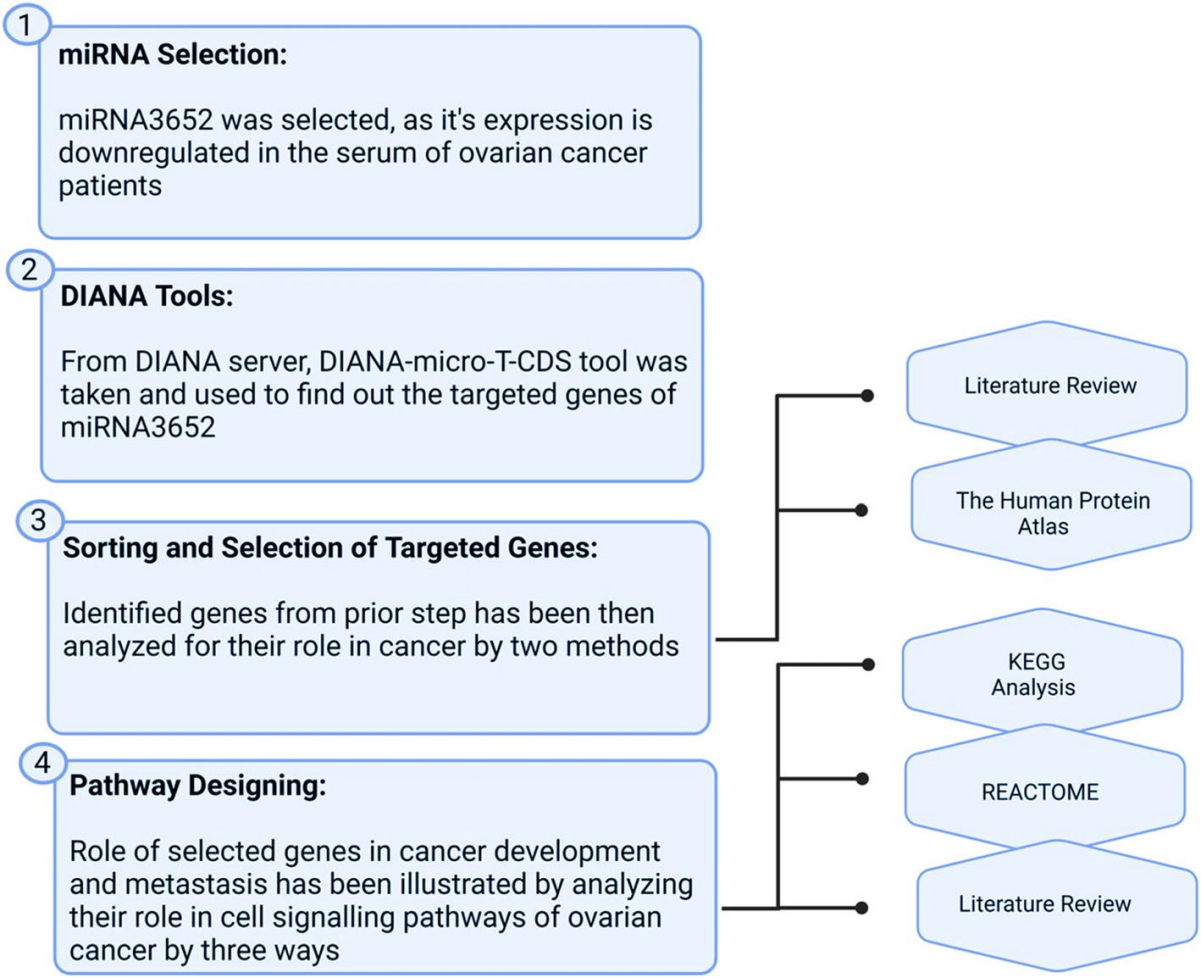
Flow diagram outlining the methodology of the study

This was a significant study choice because miRNA-3625 was downregulated in the serum and plasma of ovarian cancer patients. Additionally, its downregulation in resistant ovarian cancer cell lines due to epigenetic changes made it appear a crucial issue [43]. An exhaustive literature search on databases (Scopus and Web of Sciences) revealed that there isn’t any mechanistic study highlighting the role and/ or mechanism of miRNA-3652 in generating tumour microenvironment. The correlation between miRNA-3652 and OC was already highlighted, altering their regular expression of genes in neighbouring cells producing tumour microenvironment. The methodology has been represented by a flow sheet diagram in (Fig. 1).

### DIANA tools

The speculative identification of miRNA-3652 target genes in cells uses DIANA tools) [57] http://diana.imis.athena-innovation.gr/. An ongoing project of the DIANA-lab group for miRNA target prediction from DIANA tools, DIANA-microT-CDS (http://www.microrna.gr/microT-CDS) was chosen owing to its specificity for human beings and its machine learning approach for training data to make predictions and decisions. This programme is explicitly told to provide separate scores for the positive and negative sets of miRNA recognition elements (MREs), merging the two values into a single standard score known as the miTG score [36]. The microT-CDS tool's threshold value 0.7 was set to prevent any misleading positive or negative results. This threshold value was employed as the foundation for choosing the truly targeted genes in sensitivity analysis because of its stringent high accuracy in providing hits with a mitTG score greater than 0.7 and an increased likelihood of an accurate prediction [4].

### Assortment of targeted genes

Micro T-CDS tool-recognized genes were furthermore divided for their endowment in cancer. Two approaches were used, “The Human Protein Atlas” http://www.proteinatlas.org/) [73] which is a literature review and an online database. Extensive literature analysis was executed to disregard the genes not concerned with cancer progression and metastasis. The selected genes highlighted from the micro T-CDS tool were critically evaluated based on specific guidelines during the literature analysis; these guidelines included the gene's documented regulation in ovarian cancer, its role in targeting cancer progression signaling pathways, and publications within a time window from 2010 to 2020. By classifying genes described in research publications playing the dynamic function in many metabolic pathways in cancerous cells, genes directly engaged in the growth of cancerous cells and metastasis were concentrated for definitive selection. There is a large amount of data about all human protein-coding genes in the open-access database known as The Human Protein Atlas v.14 [74]. The Human Protein Atlas portal is divided into four sections, including photos and information on antibody-based proteomics and transcriptomics. The sections are normal tissue, cancer tissue, sub-cellular, and cell lines [55]. The Cancer Atlas (http://www.proteinatlas.org/cancer) from these subportions enfolds a mass of human cancer specimens exhibiting the 20 most related types of cancer to create protein expression profiles utilising immune histochemistry, which is why we are interested in it [7]. Using this database, the gene list was further categorised based on the expression level of human genes in cancer cells. Only the expressed genes in ovarian cancer were kept in the gene list, and others were abandoned.

### Pathway designing

The association between these genes and certain types of cancer has been elucidated by investigating the involvement of specific genes in ovarian cell biological processes. To illustrate the influence of miRNA on the targeted gene in ovarian cancer, an extensive literature analysis was conducted in conjunction with utilising two separate pathway databases, namely KEGG and Reactome. The results of pharmacological studies were employed as the source of data from the KEGG and Reactome databases. Additionally, each pathway in the KEGG and Reactome databases has been assigned a unique reaction ID, which can also be used for data regeneration.2.5 KEGG. KEGG (Kyoto Encyclopedia of Genes and Genomes) is a resource for high throughput data analysis (http://www.genome.jp/kegg/) that is open to the public [34]. KEGG was the first to offer complete pathways that were manually arranged using information from many organisms' genome projects [35]. The KEGG pathway database (http://www.kegg.jp/kegg/pathway.html) decodes how genes and molecules are crisscrossed by providing graphical information on biochemical and regulatory pathways. The KEGG pathway database offers graphical representations that elucidate the complex interactions among genes and molecules, shedding light on biochemical and regulatory mechanisms (http://www.kegg.jp/kegg/atlas.html). Each map is dynamically created using the Kegg-Sketch programme to show insights into the molecular interaction and reaction networks [21]. The KEGG Atlas is a slashing graphical interface with zooming and navigation features for the KEGG global maps. To filter organism-specific pathways connected to specific genes, we have retrieved information using the organism's name [34, 35] and utilised the KEGG DISEASE domain to explore the involvement of our specific gene in pathways associated with cancer, to narrow down our understanding. Additionally, we selected related pathway domains to identify a comprehensive list of pathways in which our particular gene is implicated, serving as entry identifiers to visualise other signalling pathways where the gene plays a crucial role. This approach strengthened and streamlined our understanding of the gene's significance and involvement in various signalling pathways.

### Reactome

Reactome is a free database, open access, thoroughly reviewed, and well-organized (http://www.reactome.org/). The Reactome database systematically links human proteins and their functions [19] to a wide range of biological processes that are expressed as a single, stable reaction pathway. Because of these networks and pathways, researchers can understand a wide range of significant disease processes at the molecular level. Information was gathered using the easy text search tool to look up processes, proteins, and pathways linked to specific genes [48]. Searched results were filtered out based on critical parameters such as molecular entity, event type, specie, cellular compartments and type of reactions. Afterwards, we further expanded our search by selecting locations in the pathway browser to visualise the organisation of events in Reactome, such as in the immune system, signal transduction, apoptosis, and many others. Ultimately, we selected event names playing a role in cancer metastasis-related to our gene to open the corresponding pathway diagram.

### Literature review

Regarding all of the targeted genes in the KEGG and Reactome databases, we found no direct involvement. By analysing the publications to have a thorough understanding of the actions of the genes in ovarian tissues, the pathways are dynamically built to address this issue.

## Results

Initially, DIANA-Micro-TCDS tools suggested that thirty-four genes were connected to miRNA-3652. Based on articles, extensive data searching of biology-integrated databases (KEGG, Reactome), and discovering that this microRNA's expression was downregulated in ovarian cancer cells, it eventually reduced to eleven genes. The concept behind the downregulation of miRNA-3652 was that the overexpression or up-regulation of the eleven genes described above would enhance the tumour microenvironment and metastasis (Table 1). The following molecular pathways have been used to describe and demonstrate how the selected eleven genes correlate with the growth and spread of ovarian cancer.

**Table 1:** Genes up-regulated by miRNA-3652 in Ovarian Cancer

**No**	**Ensemble Gene ID**	**Gene Description**	**Chr**	**Biological Process**	**Annotation source** http://www.reactome.org/	**Expression**
1	ENSG00000163660 CCNL1	Cyclin L1	3	Cyclin dependent serine/threokinase activity & cell cycle pathway	Reactome: R-HSA-156778	Ovary, Uterus, Placenta, Prostate
2	ENSG00000143190 POU2F1	POU Domain, class 2 transcription factor 1	1	Proliferation and immune modulation	Reactome: R-HSA-6807496	Ovary, Bladder, Pancreas
3	ENSG00000074047 GLI2	Zinc finger protein GLI2	2	Mediator in Hedgehog signaling pathway	KEGG: hsa04340 Reactome: R-HSA-5617413	Ovary, Prostate, Urinary bladder
4	ENSG00000112658 SRF	Serum response factor	6	RHO GTPase effectors MRTF-SRF pathway	Reactome: R-HSA- R-HSA-9625479 KEGG: nt06110	Ovary, Prostate
5	ENSG00000147862 NFIB	Nuclear Factor IB	9	Estrogen independent phenotype environment	Reactome: R-HSA-60136	Ovary, Prostate, Pancreas
6	ENSG00000160710 ADAR1	Adenosine Deaminase	1	mRNA Editing- A to I conversion	Reactome: R-hsa-77042	Intestine, Ovary, Lungs
7	ENSG00000138741 TRPC3	Short transient receptor potential channel 3	4	Calcium influx regulation	Reactome:R-hsa-426223	Brain, head, ovary
8	ENSG00000170145 SIK2	SIK2	11	PI3-AKT pathway and FA oxidation	KEGG: hsa04922	Heart, Lungs, Pancreas, Ovary
9	ENSG00000038427 VCAN	Versican gene	5	Integrin pathway and cell adhesion	KEGG: hsa04514	Liver, Heart, Muscle, Ovary
10	ENSG00000160741 CRTC2	CREB Regulated Transcription Coactivator 2	1	Up-regulation of glucose level	KEGG: hsa200186	Ovary, Prostate, Pancreas
11	ENSG00000141736 ERBB2	ERBB2 receptor tyrosine kinase 2	17	Microtubule capture and stabilization	KEGG:hsa 2064	Lung, Skin, Placenta, Ovary

### CCNL1

**Fig. 2:**
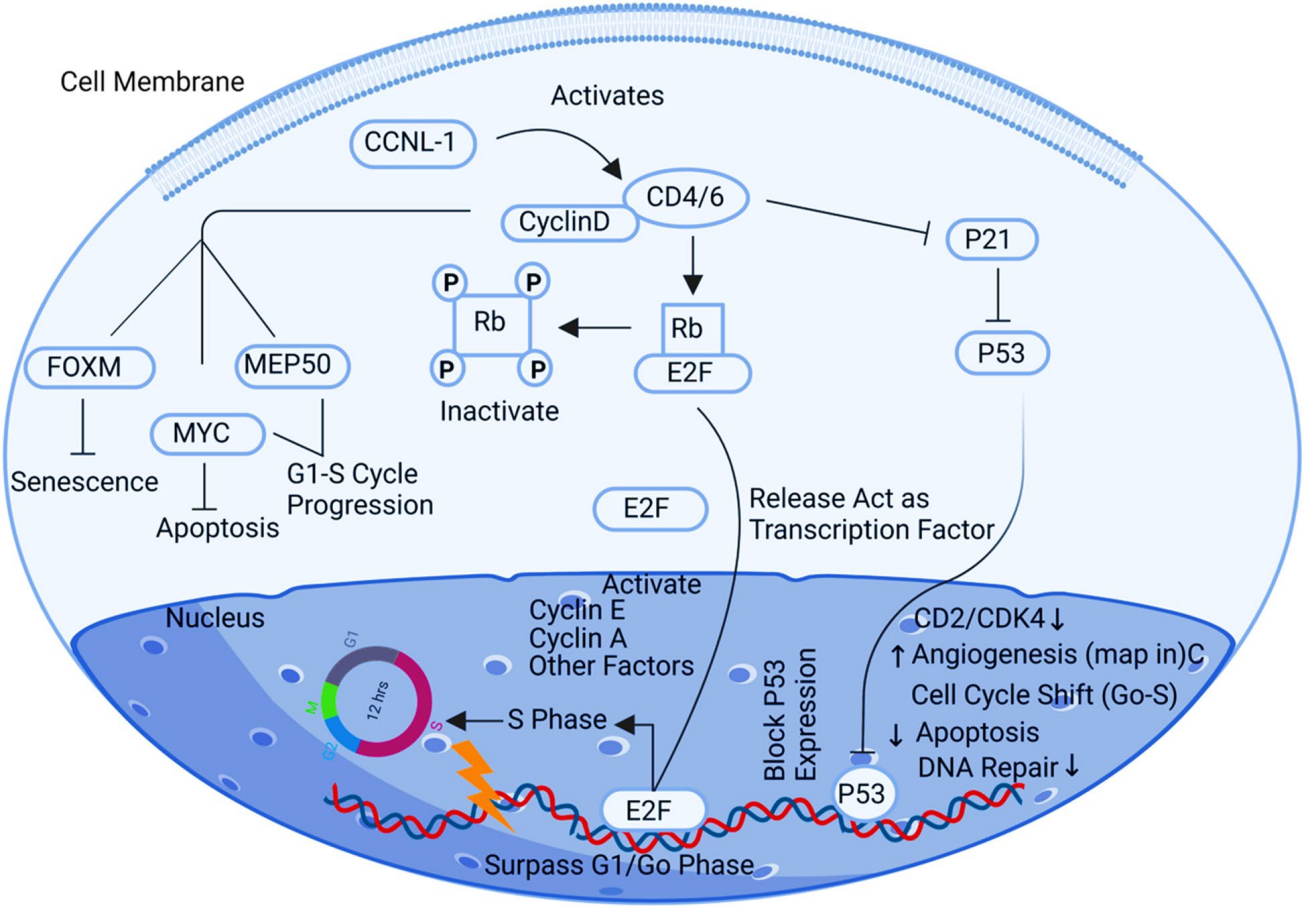
Illustration of CCNL1 signaling pathway. Positive upregulation of the CCNL1 leads to surpassing cell cycle checkpoints**,** tumour cell proliferation and genomic instability, stimulating the tumour microenvironment

The Cyclin L1 (CCNL1) gene encodes a protein that regulates cyclin-dependent serine/threokinase activity as well as the phosphorylation of the C-Terminal Domain, which is crucial for the splicing of pre-mRNA [17]. It had been noted that ovarian cancer cells overexpressed this gene. Due to this protein's high expression, ovarian cells could pass the G0 restriction point. Accordingly, the cancerous cells will produce more copies, and genomic instability will lead to tumour microenvironment [58]. By activating cyclin-dependent kinase (CDK) precisely to CDK4/6, the activity of serine/threokinase is positively regulated by Cyclin L1. Cell cycle restriction to the G0 phase [50] is related to CDK4/6 activity. When changes are established in the cellular environment developing conversion of cells to abnormal or non-mortal cells, the normal cells restrict themselves permanently to G1/G0 phase. By inhibiting the tumour suppressor proteins p21 and p53, CDK4 up-regulation promotes a cellular microenvironment that is more conducive to oncogenesis, allowing damaged cells or cellular DNA to segregate and produce more tumour cells that escape cell cycle checkpoints [1]. By forming Cyclin-CDK complexes, CDK4 also phosphorylates Rb, a tumour suppressor gene, causing it to become inactive and releasing all bound proteins, advancing tumour cells into the S phase. The proliferation of tumour cells and genomic instability is caused by the stimulation of several transcriptional factors involved in the S phase of the cell cycle, including the crucial target protein E2f, by the phosphorylation of Rb [52]. CDK 4/6 cyclin D complexes can also phosphorylate and regulate the expression of transcription factors, including FoxM1, Myc, and ME50, promoting an environment for cancer progression (Fig. 2).

### GLI2

**Fig. 3:**
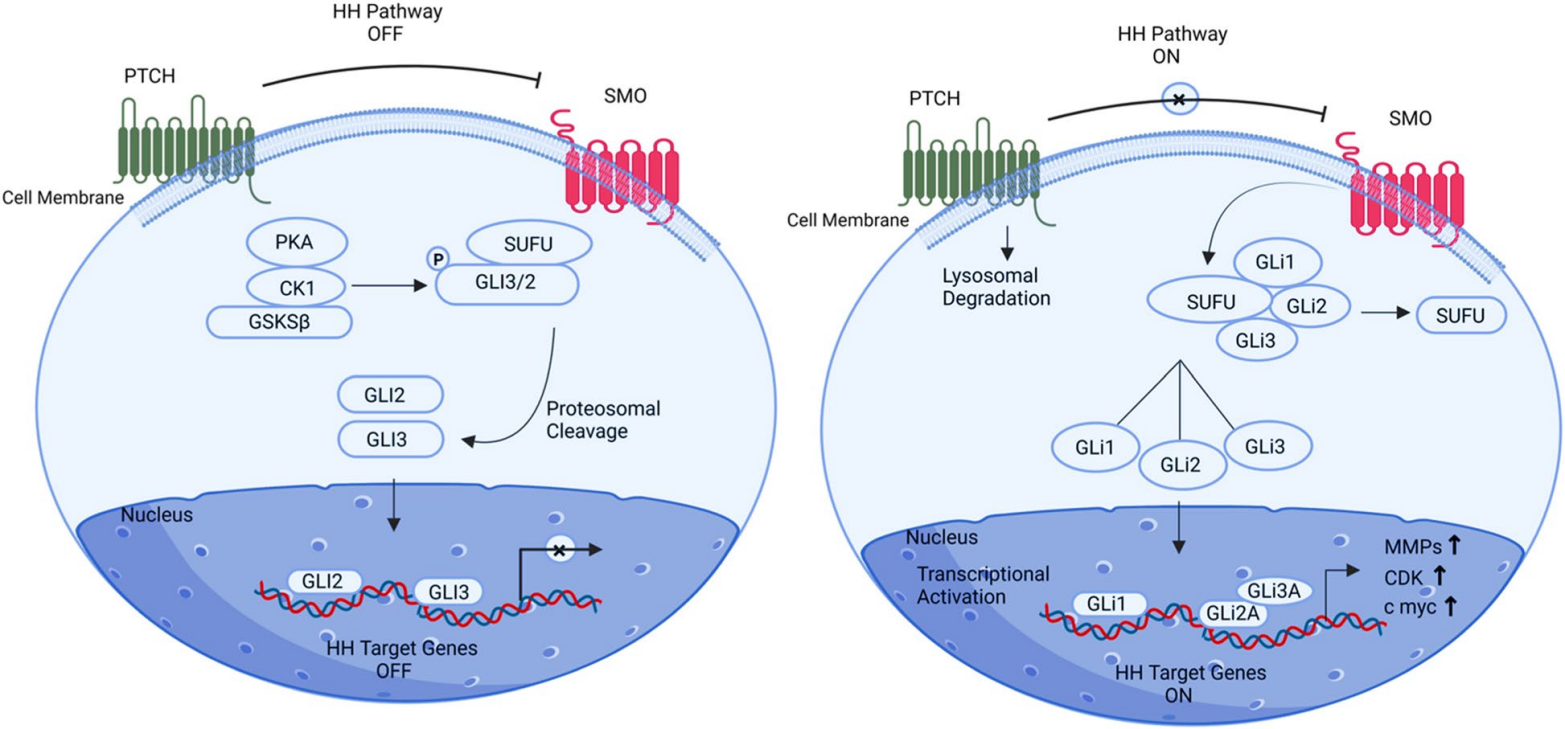
Illustration of the GLI2 signalling pathway. GLI2 acts as a moderator in the Hedgehog signalling pathway and acts as a dynamic oncogene in ovarian cancer cells; that’s why its high expression induces cellular differentiation and proliferation

Zinc finger protein GLI2 functions as a dynamic oncogene in ovarian cancer cells, causing cellular differentiation and proliferation, and as a moderator in the Hedgehog signalling pathway. The cytoplasmic GLI2 protein is related to the Hedgehog signalling pathways’ PTCH1 (Patch transmembrane) receptor [54]. Repression of SMO is lessened by allowing dissociation from SUFU, which leads to the translocation of full-length GLI2 into the nucleus and activates transcription of HH target genes, promoting ovarian cancer cell proliferation, survival, and invasiveness (starting with the activation of PTCH1 receptor onto HH ligand binding) [60] (Fig. 3). High expression and cellular trafficking of GLI2 lead to the overactivation of Hedgehog (HH)-targeted genes, thereby accelerating the progression of malignant tumors, especially in the context of ovarian cancer**.**

### NFIB

**Fig. 4:**
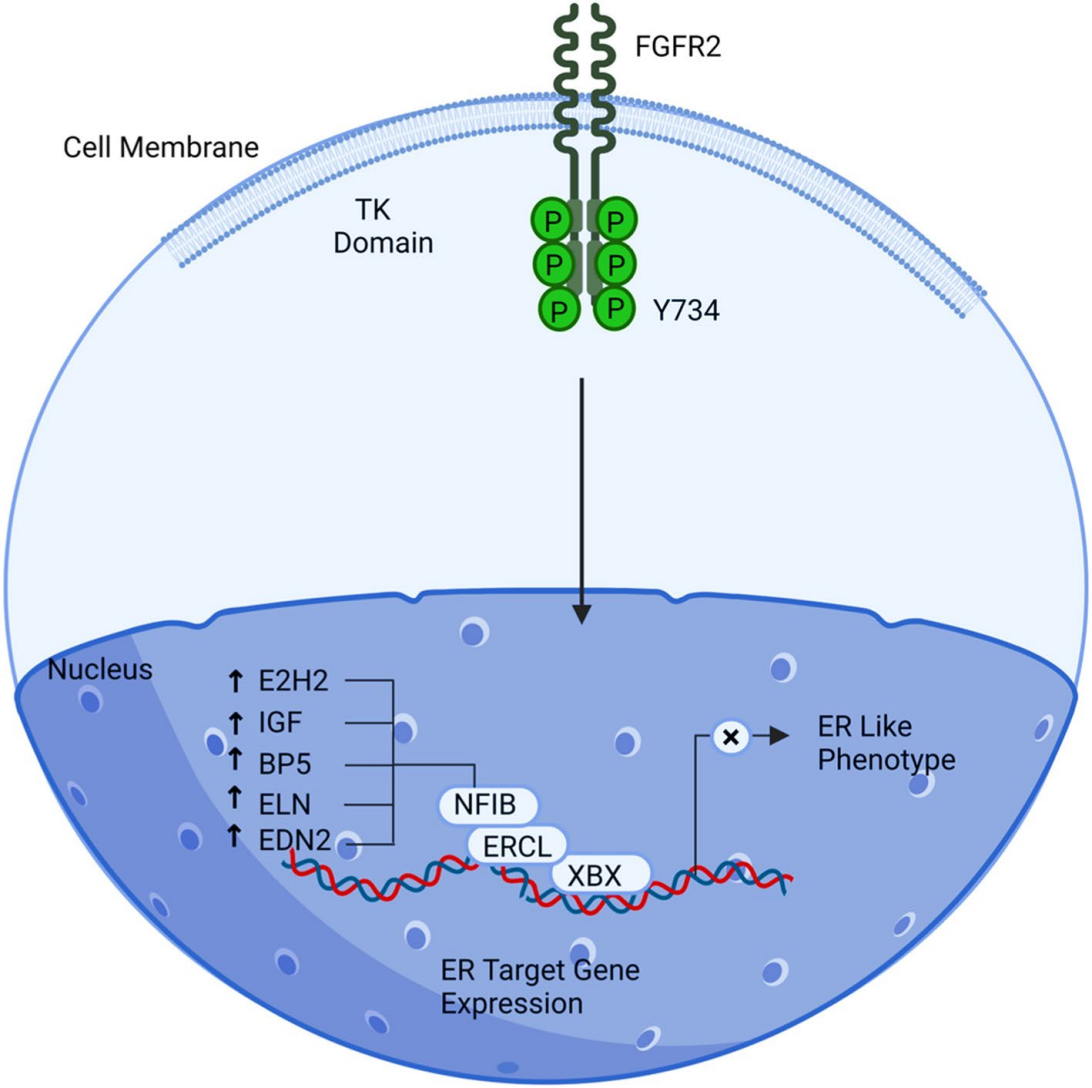
Illustration of NFIIB signalling pathway. Ovarian cancer cells exposed to an estrogen-independent environment are more likely to proliferate, behave aggressively, and have the potential to propagate

A transcriptional activator encoded by Nuclear Factor IB (NFIB) is capable of binding to other factors, including YBX1 helping in attachment with Estrogen receptor (ESR1), developing phenotype environment independent of estrogen in the ovarian cells [56]. NFIB behaves as an oncogene as it appeared to be over-expressed in ovary cells as a consequence of the down-regulation of miRNA-3652. The complex formed by NFIB, YBX1, and ESR1 suppresses ESR1 while also promoting angiogenesis, proliferation, impulsivity, and metastatic potential. Lower survival and poor prognosis are connected with the patients having repressed ESPR1 and high expression of YBX1: estrogen-dependent and independent ways provoking and amplifying tumorigenesis at the cellular level [9]. Developing reactive metabolites and forming mutagenic DNA adducts are crucial in transforming ovarian cells into cancerous tumours independent of estrogen. Additionally, the repression of ESR1, which is responsible for estrogen signalling, leads to the generation of resistance against anti-estrogen therapy. This phenomenon, known as endocrine resistance, significantly amplifies the progression of ovarian cancer and hampers the effectiveness of anti-estrogen treatments. Several factors, including EZH2, SFTPC, IGFBP5, ELN and EDN2, are regulated by NFIB, which have considerate values in the tumour microenvironment, advancing cancer impulsiveness, angiogenesis, migration and spread (Fig. 4).

## SIK2

**Fig. 5:**
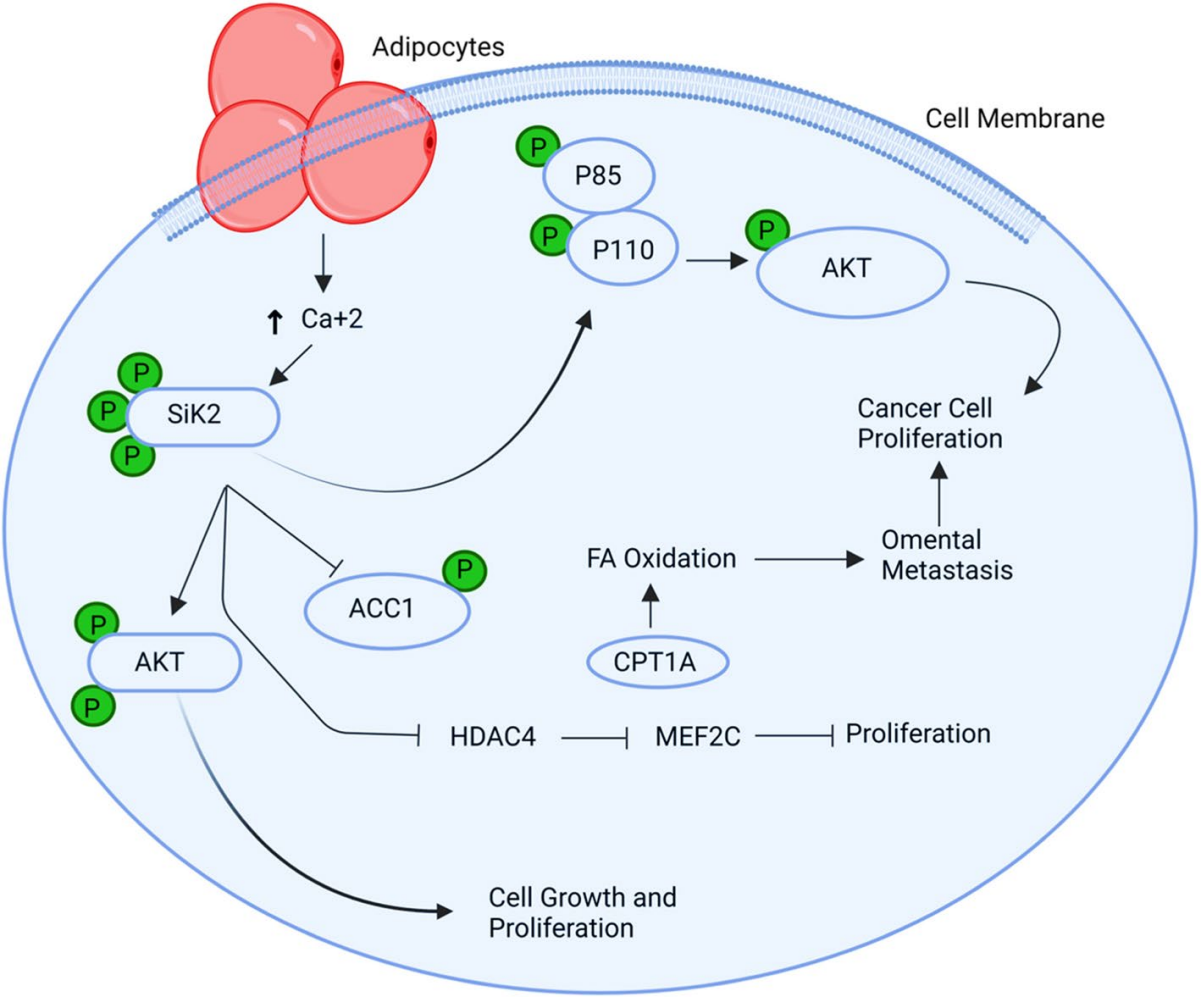
Illustration of the SIK2 signalling pathway. One of the key players in metastasis, SIK2, upregulates the PI3-AKT Pathway and FA oxidation, boosting the proliferation of ovarian cancer cells by releasing free radicals into the microenvironment of the cell and turning it into a more malignant one. SIK2: Salt-Inducible Kinase 2; PI3-AKT: Phosphoinositide 3-Kinase—Protein Kinase B; FA: Fatty Acid

One of the key players in the metastasis in the tissues (omentum and adipocyte) of the ovary that leads to HGSO (high-grade serous ovarian cancer) is the salt inducible kinase 2 (SIK2) [32]. At the location of metastasis in the body, p85 and ACC are phosphorylated to cause cell proliferation, fatty acid oxidation, and the development of the PI3-AKT Pathway inside adipocytes. It is activated due to the adipocyte's feedback of free fatty acids and by the intracellular calcium pathway. SIK2 plays a role in the phosphorylation of p85α, ACC eventually upregulates FA oxidation, and the PI3-AKT pathway stimulates the growth of ovarian cancer cells by releasing free radicals in the cell's microenvironment making the cell even more cancerous [69] (Fig. 5).

## ADAR1

**Fig. 6:**
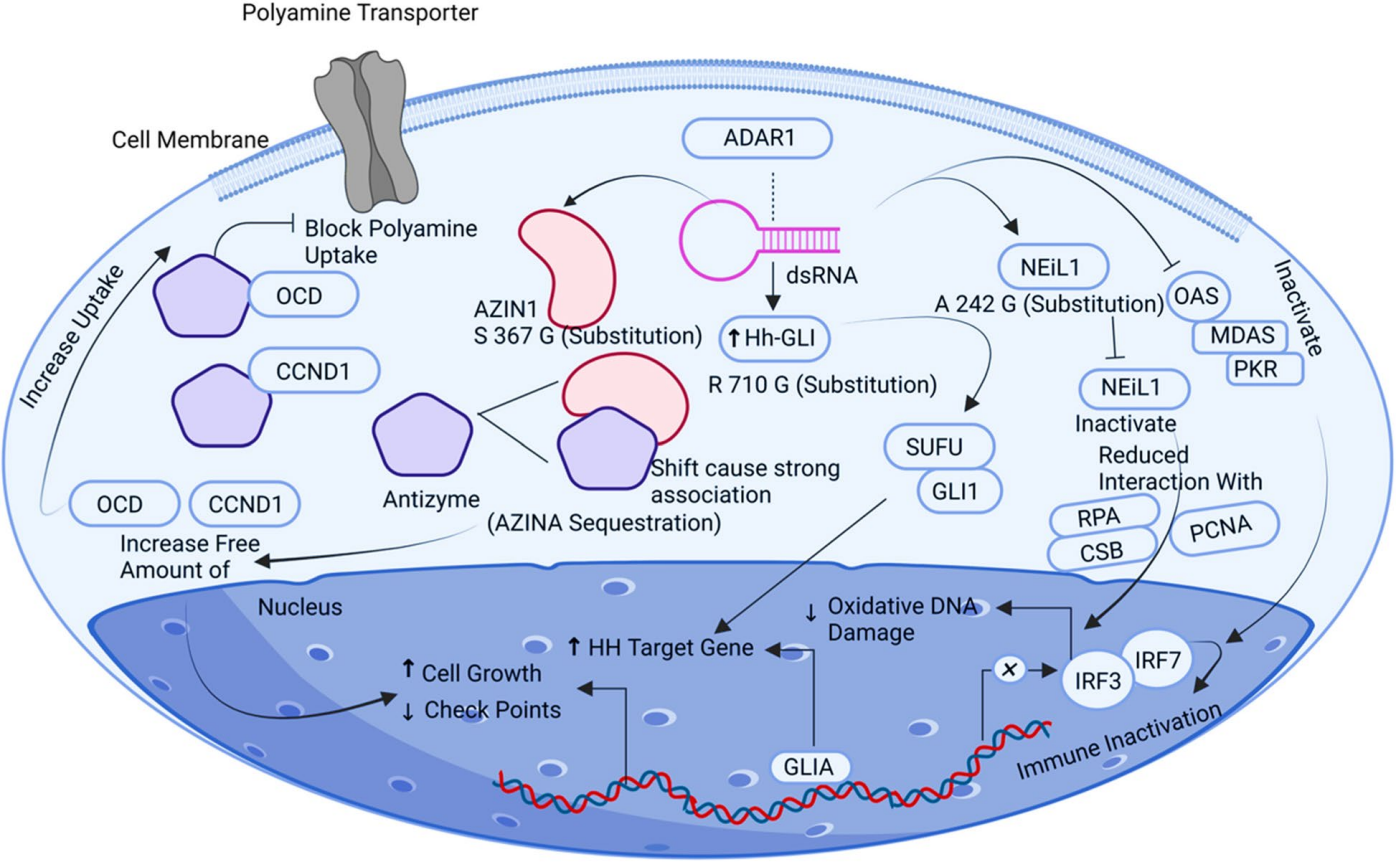
Illustrative scheme regarding the ADAR1 signalling pathway. ADAR1 editing enzyme Adenosine to Inosine in dsRNA. ADAR1 is linked to the growth of cancer through specific substrates editing in which most AZIN1 (S367G) substitution is the most usual one, GLI1 leads to R/G replacement, edited NEIL1, editing at many positions of MDA5, PKR, OAS resulting in inhibition of these proteins by triggering the inactivation of the immune system developing tumorigenesis in the cells of the ovary

An enzyme involved in A to I (adenosine to isosine) dsRNA editing is adenosine deaminase (ADAR), which acts on RNA. AZIN1 (S367G) alteration is the most prevalent one in ADAR1 substrates, which are associated with cancer progression by increasing its stability through interaction with an enzyme called antizyme and raised amounts of AZIN1 [23]. Antizyme modulates the breakdown of two important factors, ODC (ornithine decarboxylase) and CCDN1 (Cyclin D1), by having a strong link with them. Activation of ADAR1 cause an increased amount of polyamine through overexpression of ODC, enhancing polyamine transport activity in tumour cells and causing tumour cell growth. Thus, giving rise to cell growth and lowering G1/D cell checkpoint potentials clears the way for cancerous cells to enter into the cell cycle by escalating the lethal effect of cancer cells [23]. ADAR1, in conjunction with Glioma-associated oncogene-1 (GLI1), facilitates the substitution of R/G at position 701, which has been strongly associated with multiple tumours. GLI1 is well-known for its role in promoting the activation of the Hedgehog pathway, which ultimately contributes to tumour growth and progression [82]. Endonuclease 8-like 1 (NEIL1) is another substrate that undergoes hyper-editing by ADAR1, and its clinical significance in tumour development has been established. An increase in edited NEIL1 levels can significantly impact the promotion of a tumour-inducing environment. This is achieved by impairing the oxidative DNA damage repair capability, leading to an accumulation of DNA damage within the cells. The compromised DNA repair mechanism contributes to the progression of tumours and the development of a favourable environment for tumour growth. ADAR1 is also in control of (A to I) editing at many positions of MDA5, PKR, and OAS, resulting in the inhibition of these proteins by triggering the inactivation of the immune system, developing tumorigenesis in the cells of ovary [77] (Fig. 6).

## TRPC3

**Fig. 7:**
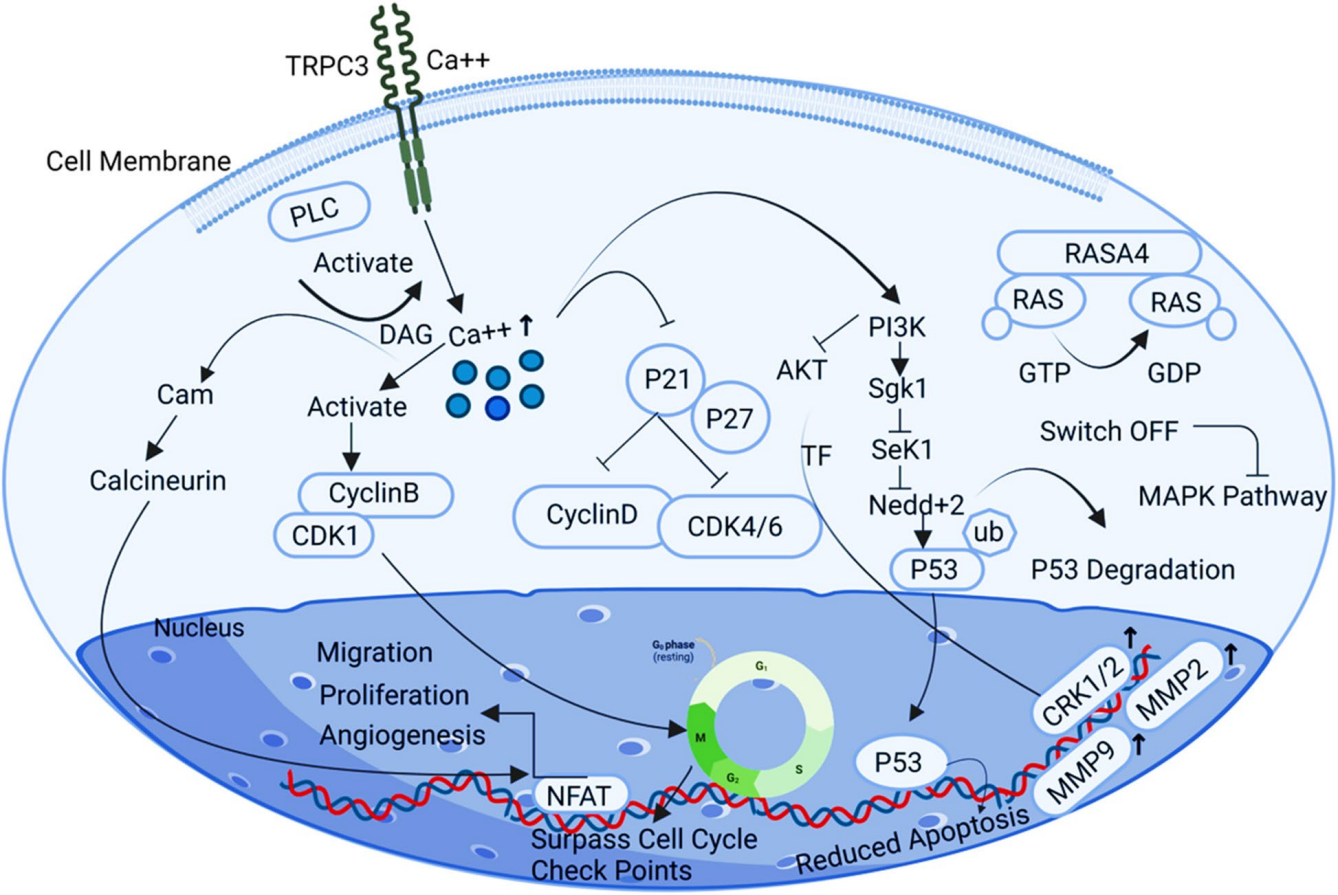
Illustration of the TRPC3 signalling pathway. TRPC3 regulates Calcium influx in the cytoplasm allowing the cell cycle to surpass checkpoint boundaries promoting the metastatic ovary microenvironment by developing tumour proliferation and increasing the stability rate

A class of diacylglycerol-sensitive cation channels known as short transient receptor potential channel 3 (TRPC3), or TRPC3, controls intracellular calcium ions by activating the phospholipase C (PLC) pathway, which is essential for preserving a balance between the processes of cell proliferation, growth, and antiapoptosis [27]. TRPC3 acts as an antiapoptotic regulator through the RAS-AT-MAP kinase pathway and is overexpressed in the plasma membrane of ovarian cancer cells. This elevated expression of TRPC3 in the plasma membrane promotes cell survival and inhibits apoptosis (programmed cell death) in ovarian cancer cells. The transition of the cell cycle between G1/S and G2/M processing the cell cycle beyond the checkpoint's boundary is another crucial function of calcium inflow in the cytoplasm. This function is accomplished by blocking p21 and p27, which in turn prevents cyclin D and CDK4/6. The cyclin B-CDK complex is successively activated by calcium influx, allowing the cell cycle to pass across checkpoint boundaries [71]. The PI3K-AKT pathway is phosphorylated by calcium signalling, which then affects transcription factor expression. This leads to enhanced production of MMP2, MMP9, and ERK1/2, which contributes to a more metastatic microenvironment in ovarian cancer by facilitating tumor growth and increasing stability rates [10] (Fig. 7).

## SRF

**Fig. 8:**
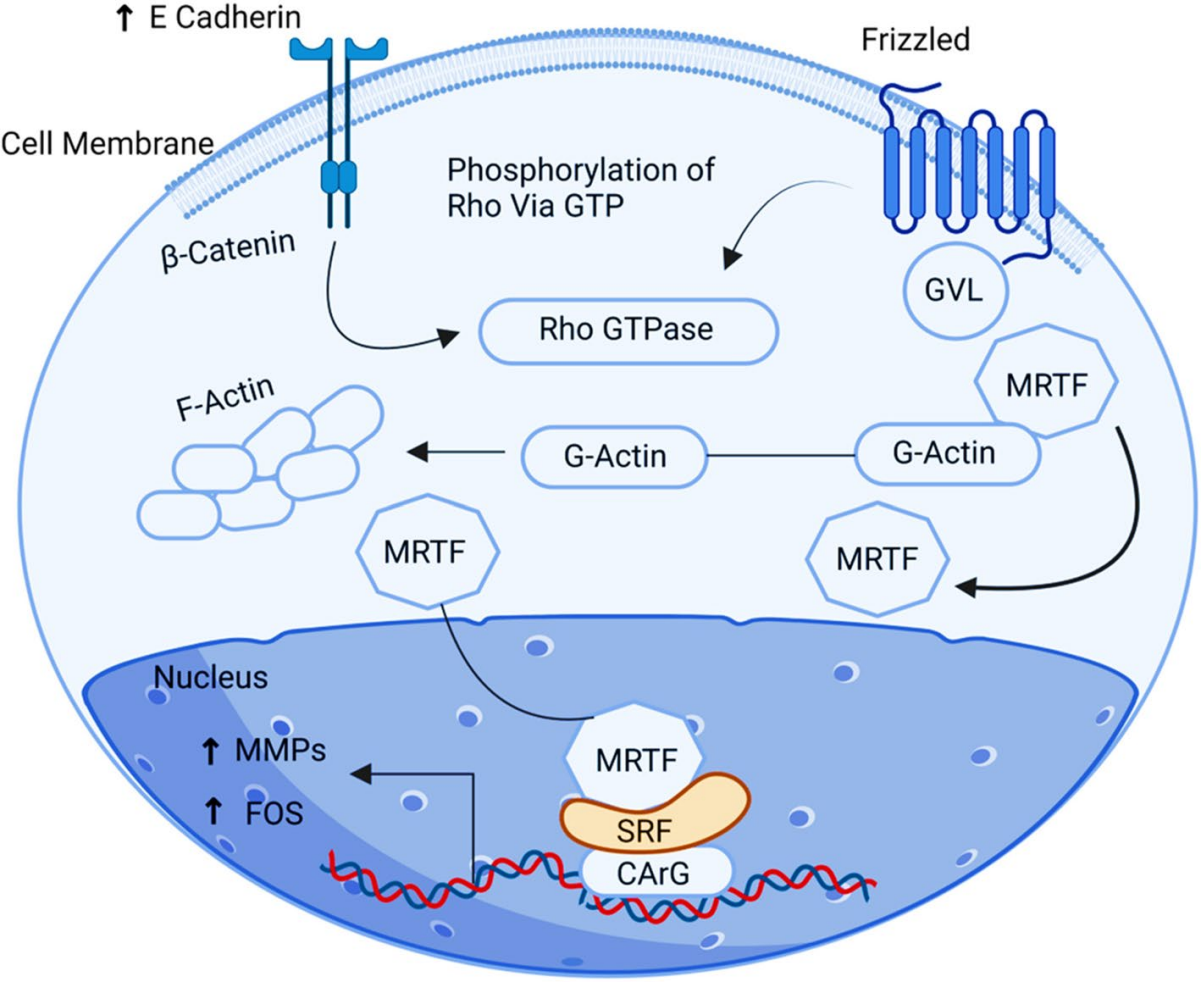
Illustration of the SRF signalling pathway. Upregulation of SRF influences elicits cell growth and proliferation through upregulation of the MRTF-SRF pathway resulting in cancer metastasis through activations of MMPs and c-Fos genes

Serum response factor (SRF) is a ubiquitous nuclear protein that binds to the serum response element (SRE) in the nucleus, which is the promoter of the targeted genes. Upregulating the MRTF-SRF pathway causes cancer spread by inducing cell growth and proliferation [47]. RhoGTPase activates MRTF by releasing G actin from it in the cell's cytoplasm. As a consequence, activation of RhoGTPase depends on the signalling of Wnt-β-catenin. POU2F1 gene accompanies the stimulation of the PI3/AKT pathway resulting in phosphorylation of the β-catenin-E-cadherin complex in the cellular environment of the ovary. This complex then activates the RhoGTPase pathway through the Wnt Signaling pathway to create a malignant environment. When MRTF releases G actin, it becomes activated and moves to the nucleus, where the SRF factor binds to the CARG Box [33]. Therefore, the MRTF-SRF complex will ultimately activate MMPs and c-Fos genes, advancing migration, growth and metastasis of ovarian cancer cells (Fig. 8).

## VCAN

**Fig. 9:**
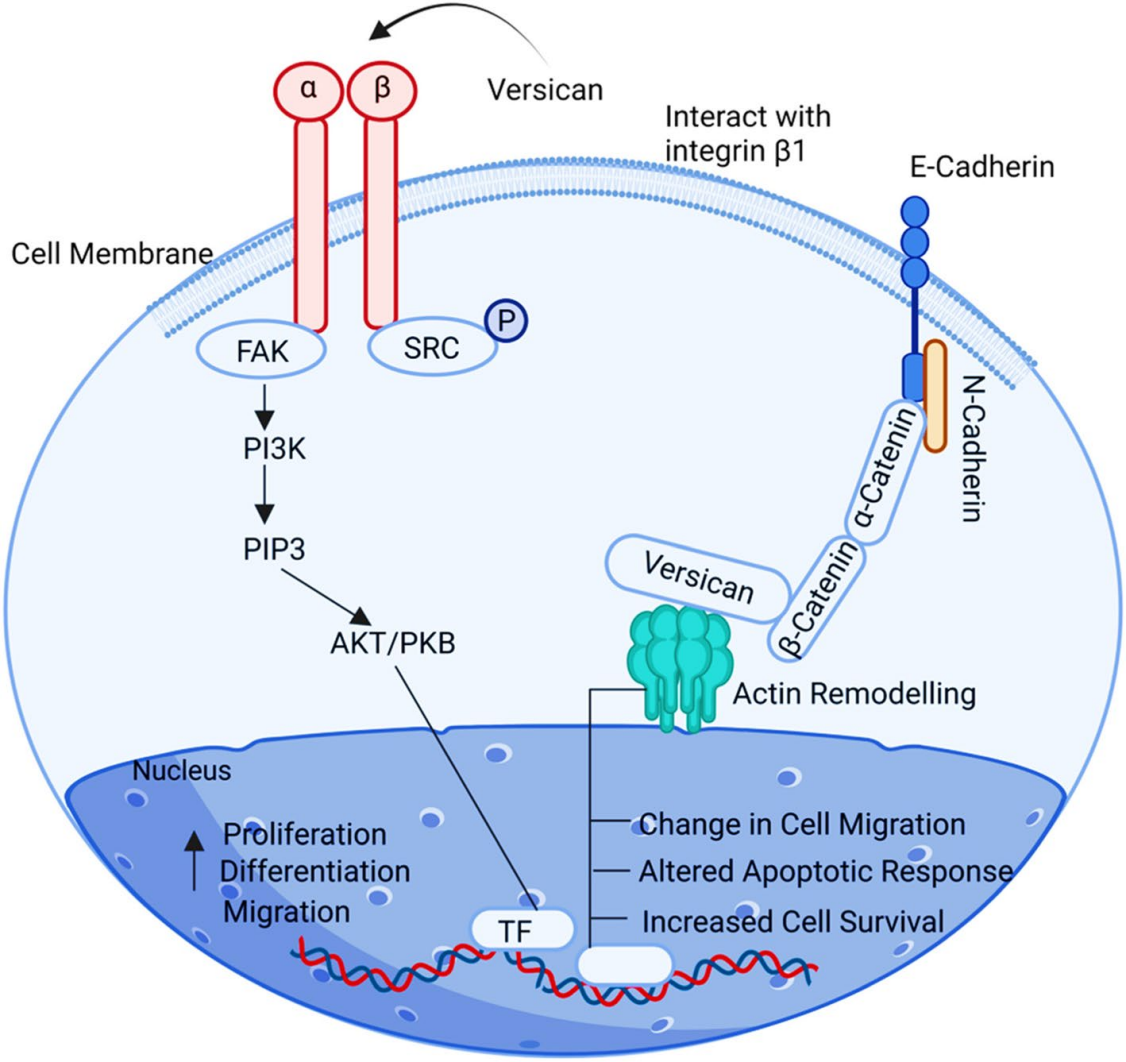
Illustration of VCAN signalling pathway. The tumour microenvironment is positively controlled by the increased expression of cancer-associated VCAN in malignant ovarian cells, which also stimulates TGF- signalling, aiding in ECM remodelling for the growth and propagation of cancer

A chondroitin sulfate proteoglycan is encoded by the Versican gene (VCAN), which plays a role in cell adhesion, proliferation, migration and angiogenesis, eventually taking part in crucial roles such as maintenance of extracellular matrix and morphogenesis of tissue. VCAN is also responsible for modulating the Wnt-mediated β-catenin signalling [76]. VCAN expression in ovarian cancer was deemed to be overexpressed. Extracellular matrix proteins have a significant role in the development of cancer. The increased expression of cancer-associated VCAN in the cancerous ovarian cells triggering the tumour microenvironment positively controls TGF-β signalling, which further assists in ECM remodelling for the advancement and metastasis of the cancer [16] (Fig. 9).

## POU2F1

**Fig. 10:**
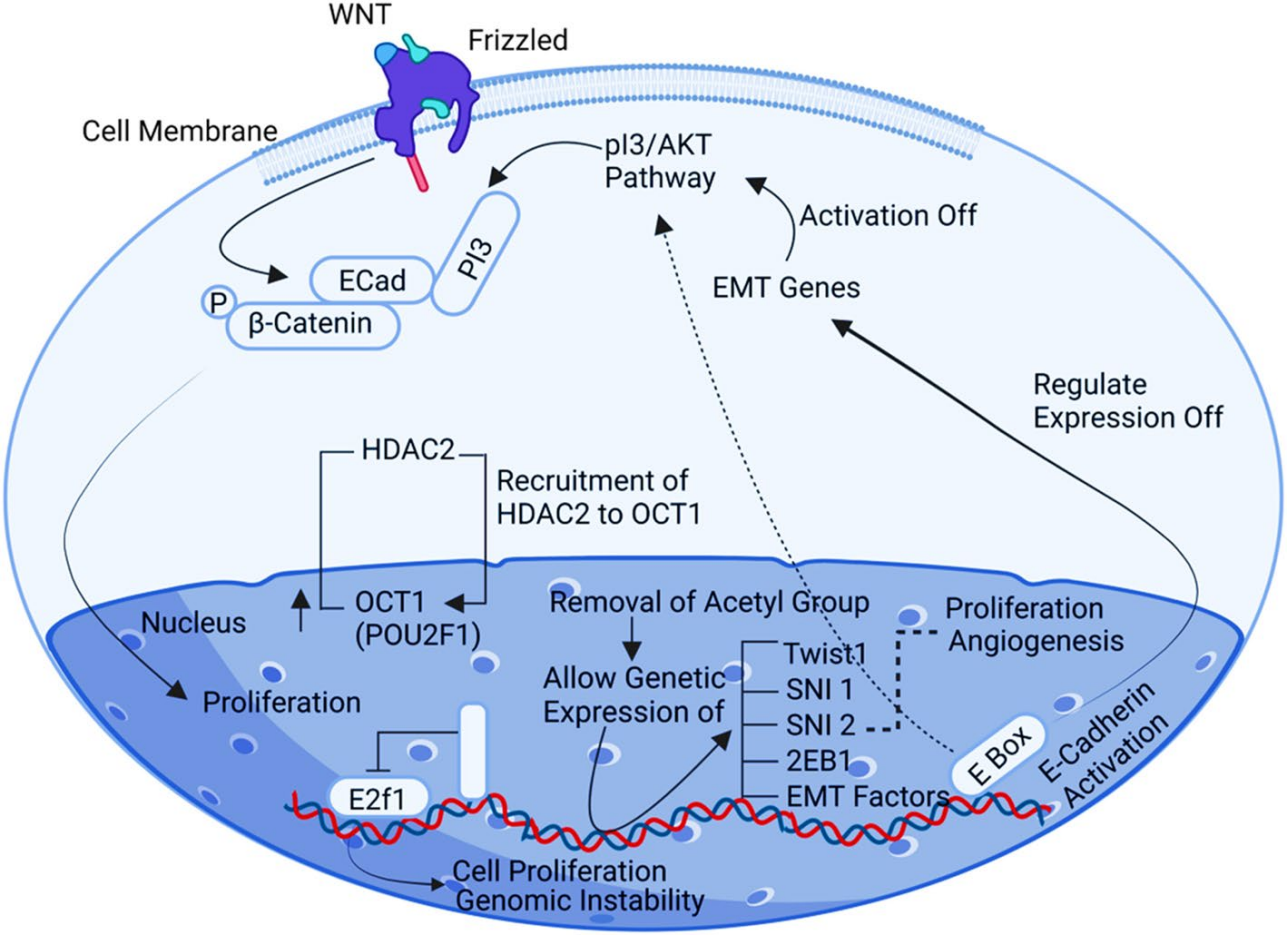
Illustration of the POU2F1 signaling pathway. High expression of POU2F1 interconnects with AKT pathways through regulation of the transcription process of EMT genes promoting cell growth, migration of EMT and invasion, acting POU2F1 as a cancerous gene

The class 2 transcription factor 1 component POU domain, which includes 160 amino acids, is required to bind an octameric sequence (ATGCAAT) [81]. The initially silent genes are stimulated and advanced by the transcription factor OCT1/POU2F1 (proliferation and immune modulation). The ovarian cancer cells express more POU2F1 when miRNA-3652 is down-regulated, which leads to growth invasion, migration, and metastasis. HDAC2 (Histone deacetylase 2) plays a key role in gene regulation by removing acetyl groups from tyrosine residues on core histones. This enzymatic activity allows for the expression of various factors, including Twist1, Snai1, Snai2, ZeB1, and EMT genes. These factors are interconnected with the AKT pathways, which are regulated during the transcription process of EMT genes. This regulation promotes cellular activities such as growth, EMT migration, and invasion. Notably, POU2F1 acts as a cancer-promoting factor in this context. Recent studies have revealed that OCT1 is upregulated in this pathway [75] (Fig. 10).

## CRTC2

**Fig. 11:**
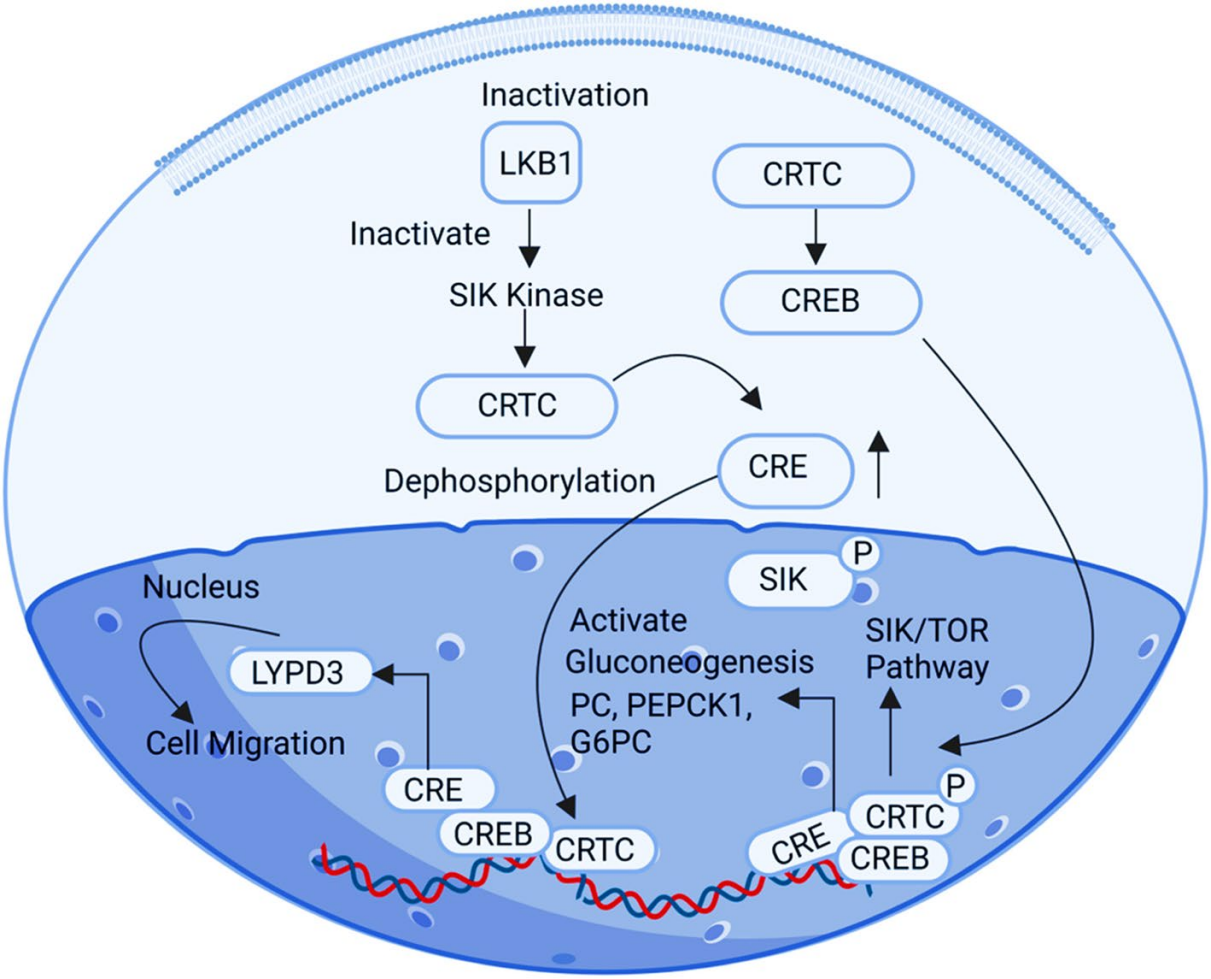
Illustration of the CRTC2 signalling pathway: when CRTC2 is overexpressed in ovarian cancer, it positively upregulates the CREB gene and promotes the overexpression of the ID1 (inhibitor of DNA binding 1) oncogene. This happens when the CREB factor binds to the ID1 promoter, amplifying ID1 expression and causing tumour growth

CRTC2 (CREB Regulated Transcription Coactivator 2) is a transcriptional co-activator that interacts with the human cAMP response element-binding protein (CREB), a transcription factor. Specifically, CRTC2 plays a role in the SIK/TORC pathway and is involved in the regulation of glucose production via the LKB1/AMPK/TORC2 pathway. Elevated expression of the CRTC2 gene may contribute to tumor development [59]. In the human body, CRTC2 binding to CREB within the nucleus is a normal physiological process involved in various cellular activities. However, oncogenesis (cell transformation) can occur under specific conditions, such as when there is irregular activity of the CREB transcription factor, which has been linked to tumor cell proliferation and antiapoptotic activity [59]. When LKB1, a tumor suppressor gene, is depleted, it results in the dephosphorylation and subsequent deactivation of salt inducible kinases. In this context, CRTC2 translocates into the nucleus and binds to CREB, leading to upregulation of the CREB gene's expression. This, in turn, promotes the overexpression of the ID1 oncogene, contributing to the development of tumors (Fig. 11).

## ERBB2

**Fig. 12:**
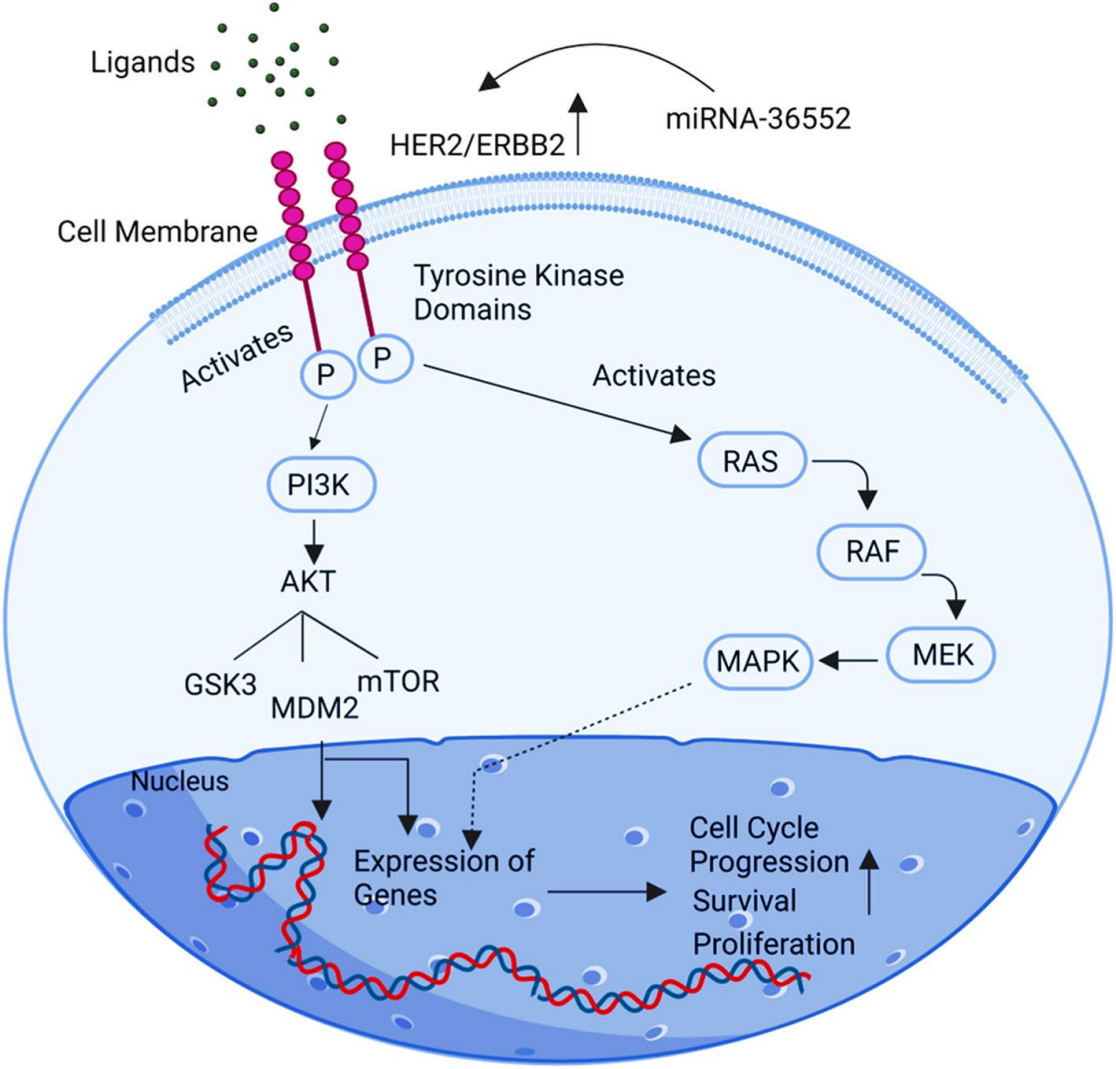
Illustration of the ERBB2 signalling pathway. Upregulation of ErBb2 can either produce metastasis-related traits like angiogenesis or invasion, or it can upregulate the curative resistance, enhancing cancer cell metastasis

Erythroblastosis oncogene B, also known as ErBb2, is a gene that essentially encodes a receptor tyrosine-protein kinase. This gene, found on the human long arm's 17th chromosome, is also known as HER2 (Human epidermal growth factor receptor 2). The epidermal growth factor receptor interacts with p185 ErBb2, a 185 kDa transmembrane glycoprotein produced by the ErBb2 gene. The ErBb2 gene's increased expression is crucial for developing tumour cells. Upregulation of ErBb2 may either produce metastasis-related characteristics like angiogenesis or invasion, or it may promote curative resistance, ultimately deteriorating cancer cell metastasis and attempting distressing responses to cancer-curative MMP-9 and MMP-2 protein activities [80]. This strategy enhances VEGF cell expression in ovarian cancer by upregulating increased ErBb2 expression, which in turn triggers a potent angiogenic response (Fig. 12).

## Discussion

### MicroRNAs: a brief overview of their normal functions

MicroRNAs (miRNAs) are a class of small, non-coding RNA molecules, usually about 20–25 nucleotides in length [3, 66]. Unlike messenger RNAs (mRNAs) that serve as templates for protein synthesis, miRNAs primarily function to regulate gene expression at the post-transcriptional level [66]. The biogenesis of miRNAs is a multi-step process [15]: i)Transcription: initially, miRNAs are transcribed by RNA polymerase II as primary miRNAs (pri-miRNAs) in the nucleus; these are long stem-loop structures; ii) Processing: the pri-miRNAs are processed by the enzyme Drosha to produce precursor miRNAs (pre-miRNAs), which are about 70 nucleotides long; iii) Export: pre-miRNAs are then exported to the cytoplasm via Exportin-5; iv) Dicer cleavage: in the cytoplasm, another enzyme, Dicer, cleaves the pre-miRNAs to produce mature miRNA duplexes; v) RISC incorporation: one strand of the duplex is incorporated into the RNA-induced silencing complex (RISC), turning it into an active miRNA, while the other strand is generally degraded [15]. The function of an individual miRNA can be context-dependent; a single miRNA can have multiple target mRNAs, and likewise, a single mRNA can be targeted by multiple miRNAs. This creates a complex regulatory network where miRNAs play pivotal roles in fine-tuning gene expression to ensure cellular homeostasis and proper response to environmental cues [31]. The normal functions of RNAs:

i) the primary function of miRNAs is to regulate gene expression; they do this by binding to complementary sequences on target messenger RNA (mRNA) transcripts, which usually results in gene silencing. Depending on the degree of complementarity between the miRNA and its target mRNA, this can lead to either mRNA degradation or inhibition of translation [67].
ii) crucial role during development and growth, they help in the precise temporal and spatial regulation of genes that drive processes like cell differentiation, growth, and the timely death of cells that have completed their roles. by repressing certain non-essential or inappropriate genes in specific cell types, miRNAs help in maintaining the unique gene expression profiles and functions of those cells [51].
iii) some miRNAs are involved in controlling the cell cycle and, thus, play roles in cell proliferation [24]; also miRNAs are involved in the regulation of programmed cell death or apoptosis, and others can influence cell survival; miRNAs can be involved in cellular responses to various stresses, including DNA damage or oxidative stress, helping cells adapt and survive or decide to undergo apoptosis [31].
iv) miRNAs play roles in various metabolic processes, including fat metabolism, insulin secretion, and more [45].
v) some miRNAs have roles in the development and response of immune cells, influencing processes like inflammation and the body's response to pathogens [12].
vi) in the nervous system, miRNAs contribute to neuronal development, plasticity, and function and they're crucial in processes like neurogenesis and synaptic plasticity [30].
vii) several miRNAs are known to influence the differentiation of hematopoietic stem cells into various blood cell lineages. Some viruses encode their miRNAs, which can interfere with host cellular functions to the advantage of the virus [64].

In physiological conditions, miRNA-3625 has been implicated in the regulation of cell cycle progression and apoptosis [42]. It is known to target several mRNAs involved in G1/S phase transition, thus maintaining cellular homeostasis [18]. Furthermore, miRNA-3625 plays a role in immune modulation by regulating T-cell activation and cytokine production [18]. Understanding these normal functions provides a baseline against which pathological changes, such as those observed in ovarian cancer, can be compared.

### Rationale behind miRNA selection in ovarian cancer studies

The selection of specific miRNAs for study in ovarian cancer is not arbitrary but is grounded in their potential biological and clinical significance [5]. By understanding the rationale behind these selections, researchers can prioritize which miRNAs to study, accelerating discoveries that might eventually translate into clinical benefits [5, 66]. In the context of ovarian cancer, certain miRNAs have emerged as central players, either as oncogenes or tumor suppressors [28, 61]. The rationale for selecting a specific miRNA for study within this malignancy often arises from a combination of factors [15, 22, 66]:

i) Differential expression in ovarian tumors: many studies rely on high-throughput sequencing or microarray analyses to determine which miRNAs are upregulated or downregulated in tumor tissue compared to normal ovarian tissue. Such differential expression can hint at a miRNA's involvement in tumorigenesis [22].
ii) Links to clinical outcomes: some miRNAs are associated with particular clinical outcomes in ovarian cancer patients, such as overall survival, disease recurrence, or response to therapy; studying these miRNAs can provide insights into disease progression and prognosis [5, 22, 66].
iii) Role in key signaling pathways: ovarian cancer progression involves several critical signaling pathways. miRNAs known to modulate these pathways, such as the PI3K/Akt pathway or the p53 signaling pathway, become key candidates for research [22, 66].
iv) Evidence from other cancer types: a miRNA's established role in other malignancies might suggest a potential role in ovarian cancer, warranting investigation [22, 66].
v) Functional impact on cellular processes: miRNAs that influence essential cellular processes like cell proliferation, apoptosis, angiogenesis, or metastasis are of inherent interest. Their dysregulation can shed light on the mechanisms behind ovarian cancer development and progression [22, 66].
vi) Potential for therapeutic modulation: miRNAs that can be feasibly targeted, either to inhibit (in the case of oncomiRs) or enhance (for tumor suppressor miRNAs) their activity, are particularly attractive for research, given the potential translational applications [22, 66].

Several studies have demonstrated a correlation between dysregulation of miRNA-3652 and ovarian cancer. It has been observed that miRNA-3652 is downregulated in ovarian cancer patients' serum, plasma, and urine. However, the exact mechanisms and processes triggered by the dysregulation of miRNA-3652 in ovarian cancer patients remain unknown. Further research is required to unravel the specific consequences and underlying pathways associated with the dysregulation of miRNA-3652 in ovarian cancer patients [28]. In this study, the researchers investigated miRNA-3652’s system of likely target genes and its potential to cause cancer in OC cells. These mutagenic effects may create a tumour microenvironment, which could have a variety of carcinogenic outcomes [70]. CCNL and ADAR1 have been disclosed to be counted in the modulation of the cell cycle. Numerous studies have confirmed that cell cycle checkpoints and cell cycle arrest are effective cell defence mechanisms to stop the progression of the cell cycle when it comes into contact with any type of genomic instability or mutagenic conditions in a cell [50, 77]. By driving cells past the G1/G0 restriction point and into the S phase, positive regulation of CCNL and ADAR1 allows the cell cycle to progress, leading to the proliferation of tumour cells and genetic instability in the tumour-producing environment. Our findings suggest that in OC, POU2F1 promotes tumour cell proliferation, EMT migration, and invasion. The expression of EMT genes aids in the migration of EMT in ovarian cells and initiates proliferation and immune regulation when POU2F1 is upregulated, which triggers tumour promoter factors that impair the immune system's health [75]. Because miRNA3652 is downregulated in ovarian cancer cells, several signalling cascades, including the WNT—catenin signalling route, the MRTF-SRF pathway, and the Hedgehog signalling system, are stimulated, leading to the overexpression of the GL2, SRF, and VCAN genes. Numerous studies have demonstrated that GL2 influences other cellular cascades by positively regulating the Hedgehog signalling pathway (e.g., cellular differentiation, proliferation, cell survival and invasiveness). As a result, GL2 overexpression may affect how OC develops. We demonstrate that increased MRTF-SRF and Wnt—catenin signalling improves the carcinogenic environment by enhancing cell adhesion, proliferation, migration, and angiogenesis in ovarian cancer cells [16, 47, 54]. Therefore, activation of the Wnt signalling pathway confirms the role of miRNA3652 in the progression of ovarian cancer. Because miRNA3652 is downregulated in ovarian cancer cells, NFIB and SIK2 are increased, which causes ovary cells to produce reactive metabolites, mutagenic DNA adducts, and free radicals [56, 69]. The dysregulated expression of these genes disrupts normal cellular function, leading to the transformation of ovarian cells into tumour cells characterised by enhanced proliferation, aggressive behaviour, and progression within the tumour microenvironment, ultimately resulting in the development of more malignant cells. These recent findings provide additional evidence to support the conclusion that reactive metabolites, mutagenic DNA adducts, and free radicals play a critical role in the persistence and metastasis of cancer cells. In OC cells, the TRPC3 enhances malignant cells' persistence and carcinogenic potential. Overexpression of TRPC3 modulates the calcium signalling pathway as it is associated with cell growth, multiplication and anti-apoptosis in ovary cells advancing the ovary microenvironment to convert into a cancerous one [71]. Calcium signalling triggers the activation of the cyclin B-CDK complex sequentially, facilitating the passage of the cell cycle through checkpoint boundaries. Additionally, the upregulation of TRPC3 promotes the activation of the PI3K-AKT pathway, which contributes to ovarian cancer progression by enhancing tumour takeover, durability, and advancement. Furthermore, our data suggested CRTC2 and ERBB2 behaved as oncogenes in ovarian cancer. The production of CRTC2 helps in the upregulation of glucose levels in the body because of the stimulation of the signalling pathway known as LKB1/AMPK/TORC2. The overexpression of CRTC2 triggers cancer cell proliferation and metastasis. At the same time, over-activating the ErBb2 gene triggers cancer cell metastasis and shows the anti-apoptotic behaviour of cancerous cells. This gene activates two other genes simultaneously: PI3k and RAS. PI3k activates AKT and blocks the expression of PTEN, while RAS activates MAPK and ERk1/2 expression and triggers angiogenic and oncogenic expression of cells [59, 80]. Investigating the pathway involving these eleven targeted genes provides valuable and conclusive insights into the cancer-promoting potential of miRNA3652 in ovarian cancer cells. The approach taken in this analysis is well-regulated and reliable. This analysis offers a comprehensive understanding of the fundamental cellular mechanisms and signalling pathways involved in the development and malignancy of ovarian cancer. The findings presented above will contribute to the future design of novel medical interventions and assessments for ovarian cancer, with enhanced treatment efficacy.

### Downregulation of miRNA in ovarian cancer

The aberrant expression of microRNAs (miRNAs), both upregulation and downregulation, is a hallmark of many cancers, including ovarian cancer; the downregulation of certain miRNAs can promote oncogenesis and cancer progression according to the following mechanisms [3, 5, 37, 44]:

i) Some miRNAs act as tumor suppressors, meaning their normal function is to prevent uncontrolled cell growth or induce apoptosis; downregulation of these miRNAs can lead to unregulated growth and division of cancer cells. In ovarian cancer, examples include the Let-7 family, miR-34, and miR-31, which when downregulated, contribute to increased proliferation, invasion, and reduced apoptosis of cancer cells [5].
ii) Genomic regions containing tumor-suppressor miRNAs can undergo deletions or mutations, leading to reduced expression of the miRNA [6].
iii) DNA methylation and histone modifications can silence miRNA gene expression; for instance, hypermethylation of promoter regions of tumor suppressor miRNAs can inhibit their transcription, leading to downregulation [63].
iv) Certain transcription factors that positively regulate miRNA expression might be downregulated or mutated in cancer; their absence or reduced activity can lead to decreased miRNA expression. For example, the transcription factor p53, often mutated in cancers, regulates the expression of various miRNAs. Any disruption to p53 can thus influence miRNA expression levels [68].
v) The biogenesis of miRNA involves a series of processing steps involving multiple proteins, including Drosha and Dicer; alterations or reductions in the levels of these proteins can lead to reduced miRNA maturation and thus downregulation. In ovarian cancer, reduced expression or altered function of Dicer has been associated with advanced tumor stage and poor prognosis [37].
vi) Competitive Endogenous RNAs (ceRNAs): ceRNAs, such as long non-coding RNAs (lncRNAs), can act as sponges for miRNAs, reducing their availability to target mRNAs. Increased expression of certain ceRNAs in ovarian cancer can thus lead to functional downregulation of specific tumor suppressor miRNAs [37].
vii) Cellular signaling pathways often have feedback mechanisms: a downregulated miRNA might target a protein that, when upregulated, further suppresses the miRNA's expression, creating a feedback loop that further reduces the miRNA's levels [44].

The downregulation of miRNAs in ovarian cancer can be a result of various genetic, epigenetic, and cellular mechanisms. Understanding these intricate pathways and regulatory mechanisms can provide insights for therapeutic interventions, aiming to restore the expression of tumor suppressor miRNAs or inhibit the pathways causing their downregulation.

### Translational studies related to miRNA in cancer therapies: focus on ovarian cancer

MicroRNAs (miRNAs) have emerged as essential regulators of gene expression, influencing a plethora of cellular processes, including proliferation, differentiation, and apoptosis [3]. In the context of cancer, aberrant miRNA expression can drive tumorigenesis, progression, and resistance to therapies, as such, miRNAs offer a promising avenue for therapeutic intervention, especially in cancers like ovarian cancer, where there is a dire need for more effective treatments [3, 15]. This section will shed light on the translational studies focused on leveraging miRNAs in ovarian cancer therapies:

i) diagnostic and prognostic biomarkers: several miRNAs have been identified as potential biomarkers for the early detection of ovarian cancer or for predicting disease outcome. For example, miR-200 family members are typically downregulated in epithelial ovarian cancer and have been linked to disease progression and chemotherapy response [15].
ii) therapeutic targets: oncomiRs—some miRNAs are overexpressed in ovarian cancer and contribute to tumorigenesis. These "oncomiRs" can be targeted for therapeutic silencing [15]; for example, miR-21 is upregulated in many cancers, including ovarian cancer, and has been associated with decreased apoptosis and increased chemotherapy resistance. AntagomiRs or locked nucleic acids (LNAs) targeting miR-21 have been explored to inhibit its function [15].
iii) tumor suppressor miRNAs: some miRNAs act as tumor suppressors and are downregulated in cancers. Restoring their expression can inhibit tumor growth. For example: Let-7 is often downregulated in ovarian cancer, synthetic Let-7 mimics have been developed to restore its function, subsequently inhibiting tumor growth.
iv) miRNA replacement therapy: involves introducing synthetic miRNA mimics into cells to restore the function of downregulated tumor suppressor miRNAs. Clinical trials have been initiated for some of these mimics in various cancers, though the challenge remains in effective delivery to tumor cells without off-target effects.
v) miRNA inhibition therapy: For oncomiRs that are overexpressed in cancer cells, antagomiRs or other miRNA inhibitors can be introduced to inhibit their function; challenges here also include specificity, delivery, and potential off-target effects [15].
vi) chemotherapeutic drug resistance: miRNAs have been implicated in chemotherapy resistance in ovarian cancer; for instance, miR-214 induces cisplatin resistance by targeting the PTEN/Akt pathway [41]. Therapeutically targeting such miRNAs can potentially resensitize tumors to chemotherapy.
vii) a significant challenges in miRNA-based therapy is effective delivery to tumor cells; diverse nanoparticles loaded with miRNAs and are being explored for targeted delivery with minimal side effects [46].

The translation of miRNA research into tangible therapies for ovarian cancer is an exciting frontier in oncology; while challenges persist, particularly concerning delivery and specificity, the potential benefits are substantial. Continued research and innovation promise to usher in a new era of targeted, effective treatments for ovarian cancer based on miRNA modulation.

## Conclusion and future perspectives

This study sheds significant light on the functional roles of miRNA-3652 in the progression of ovarian cancer. Our findings suggest that miRNA-3652 has noteworthy effects on cellular processes relevant to the malignancy, which makes it a potential biomarker and therapeutic target. Specifically, alterations in the expression of miRNA-3652 are closely linked to various hallmarks of ovarian tumor progression, including cell proliferation, migration, and apoptosis. The implications of these findings are paramount, especially considering the current gaps in understanding and treating ovarian cancer effectively. The present study underscores the functional relevance of miRNA-3652 in ovarian cancer progression, further in-depth mechanistic studies and comprehensive understanding of the molecular mechanisms by which miRNA-3652 exerts its effects will elucidate the broader role of microRNAs in cancer biology. Given the potential significance of miRNA-3652 in tumor progression, its utility as a therapeutic target should be rigorously examined, this includes exploring targeted delivery mechanisms and ensuring the specificity of the therapeutic interventions to minimize off-target effects. The potential of miRNA-3652 as a prognostic marker also should be further evaluated in clinical settings. Studies assessing the correlation between miRNA-3652 levels and clinical outcomes, including treatment response and patient survival, will be of immense value. It is essential to explore if the roles of miRNA-3652 observed in ovarian cancer are replicated in other cancer types this would allow for the exploration of common microRNA-mediated pathways in cancer and the development of more broad-spectrum therapeutic interventions. As our understanding of miRNA-3652 grows its potential synergy with existing therapeutic agents or strategies should be assessed, combining miRNA-based interventions with current therapies might offer enhanced therapeutic outcomes for ovarian cancer patients. In conclusion, this study has laid the groundwork for a deeper understanding of the role of miRNAs in ovarian cancer; a multidisciplinary approach that combines molecular biology, clinical research, and therapeutic design will be vital in harnessing the potential of miRNA-3652 in the battle against this devastating disease.

## Data Availability

Not applicable.
